# Horizontal gaze palsy with progressive scoliosis: a case report with magnetic resonance tractography and electrophysiological study

**DOI:** 10.1186/s12883-018-1081-9

**Published:** 2018-05-29

**Authors:** Chi-Wei Lin, Chung-Ping Lo, Min-Chien Tu

**Affiliations:** 1 Department of Neurology, Taichung Tzu Chi Hospital, Buddhist Tzu Chi Medical Foundation, Taichung, Taiwan; 2 Department of Radiology, Taichung Tzu Chi Hospital, Buddhist Tzu Chi Medical Foundation, Taichung, Taiwan; 3 School of Medicine, Tzu Chi University, Hualien, Taiwan

**Keywords:** Horizontal gaze palsy with progressive scoliosis, Magnetic resonance imaging, Split pons sign, Diffusion tensor imaging, Tractography, Somatosensory evoked potential

## Abstract

**Background:**

Horizontal gaze palsy with progressive scoliosis (HGPPS) is a rare autosomal recessive congenital anomaly characterized by horizontal gaze limitation and progressive scoliosis. We investigated the underlying pathogenesis by incorporating diffusion tensor imaging and an electrophysiological study.

**Case presentation:**

A 55-year-old female patient presented to our clinic due to a chronic history of eye movement limitation since childhood. Her eye problem was followed by a progressive scoliotic change in her torso during junior high school. Neurological examinations revealed remarkable conjugate horizontal but not vertical gaze palsy. Her pupils were isocoric, with a prompt response to light reflex and convergence. Her vision, including visual acuity and field, were normal. No pathological signs of muscle tone, muscle power, deep tendon reflex or coordination were revealed. There was no associated family history, and no diseases involving other systems were noted. On reviewing her past medical history, X-rays revealed scoliotic changes of her thoracic and lumbar spine. Brain magnetic resonance imaging showed a midline cleavage at the tegmentum (split pons sign) and butterfly configuration of the medulla, consistent with HGPPS. Color-coded diffusion tensor imaging in our patient revealed absence of decussation of the superior cerebellar peduncle. In tractography, the pontocerebellar tracts and fibers within the inferior cerebellar peduncle, deemed to be primarily dorsal spinocerebellar and vestibulocerebellar tracts, appeared to be agenetic. The tegmentum was compromised secondary to dorsal displacement of the corticospinal tracts. Of note, the bilateral corticospinal tracts remained uncrossed at the level presumed to be the pyramidal decussation. A somatosensory evoked potential study also revealed predominantly ipsilateral cortical sensory responses.

**Conclusions:**

Our study confirmed that a compromised tegmentum secondary to dorsal displacement of the corticospinal tracts and poorly-developed afferent fibers within the pontocerebellar tracts and inferior cerebellar peduncle to be the main neuroanatomical anomalies responsible for the clinical presentations of HGPPS. In addition, the uncrossed nature of the majority of pyramidal and proprioceptive sensory systems was confirmed.

**Electronic supplementary material:**

The online version of this article (10.1186/s12883-018-1081-9) contains supplementary material, which is available to authorized users.

## Background

Horizontal gaze palsy with progressive scoliosis (HGPPS) is a rare congenital disorder with autosomal recessive inherence, characterized by impaired conjugate horizontal eye movements and progressive scoliosis developing in childhood and adolescence [1, 2]. Genetic studies underpin that the pathogenesis of HGPPS is associated with mutations of the *ROBO3* gene, as the ROBO3 protein determines axon path finding, crossing, and resultant hindbrain morphogenesis in human [3]. The responsible pathoanatomical findings remain inconsistent; however it is generally believed to be the consequence of cranial nuclear maldevelopment, as evidenced by brainstem hypoplasia in neuroimaging studies [1, 4]. Although several conventional brain magnetic resonance imaging (MRI) studies have described its anatomical features and clinical relevance [1, 4], studies incorporating diffusion tensor imaging (DTI) tractography [5, 6] and electrophysiological assessment [6, 7] are limited. Herein, we describe a case with clinical-radiological features typical for HGPPS, and further explore its relevant pathogenesis on a neuroanatomic basis.

## Case presentation

**Fig. 1:**
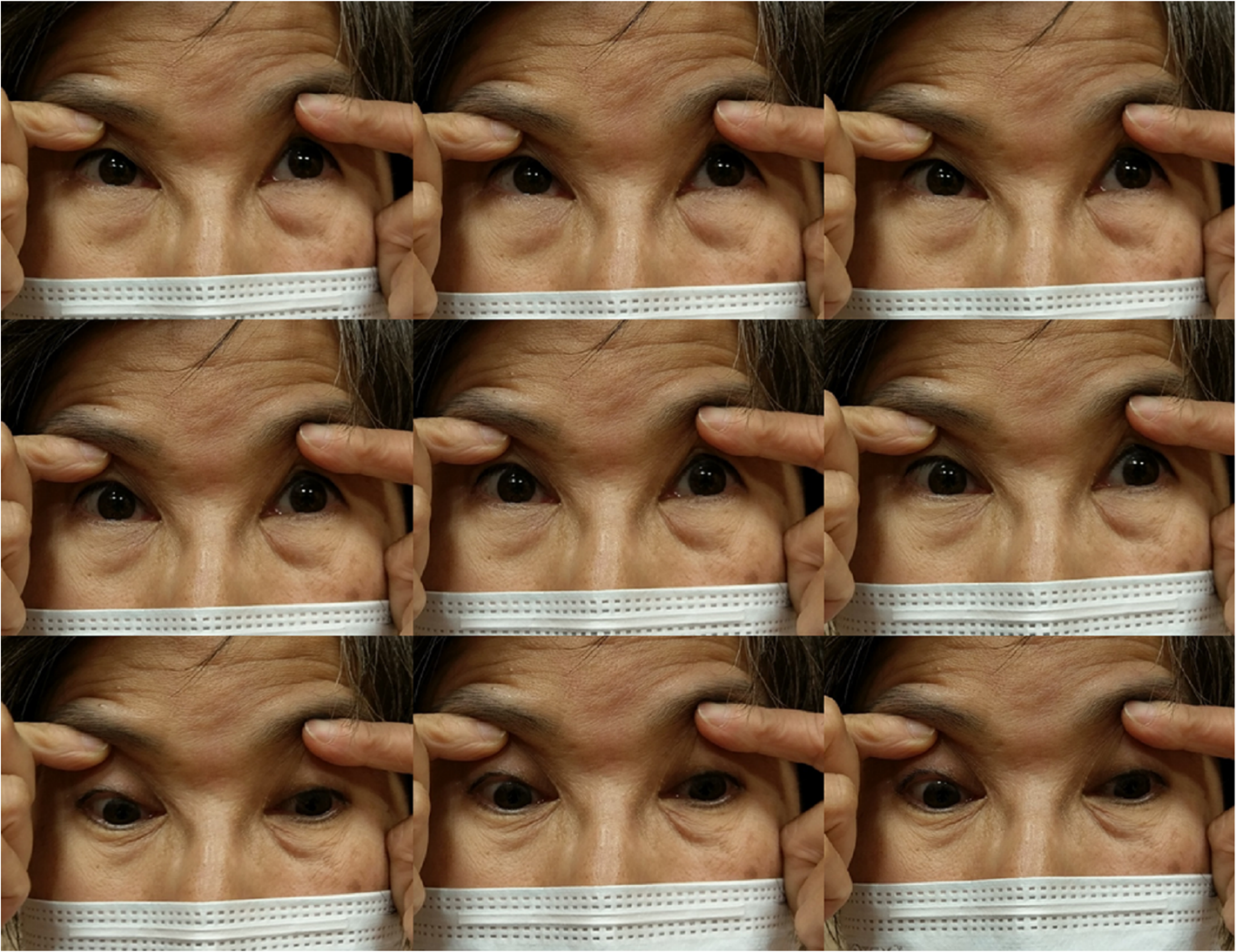
Cardinal directions of gaze in this patient. There was a limited range of voluntary ductions in horizontal directions. Vertical gazing was normal


A 55-year-old female patient presented to our clinic due to a chronic history of eye movement limitation since childhood. She had difficulty in moving her eyes to the right or left, and therefore relied heavily on turning her neck. This debilitating condition was followed by progressive scoliotic changes in torso during junior high school. Her cognition was intact, and she had no history of seizures or vision loss. Neurological examinations revealed remarkable conjugated horizontal but not vertical gaze palsy (Fig. 1) (Additional file 1). Although she was fully cooperative, saccade and pursuit movements were rarely completed. Her vestibular-ocular reflex was absent, and her pupils were isocoric with a prompt response to light reflex and convergence. No nystagmus, ptosis, chemosis, periorbital erythema, or extraocular muscle synkinesis were observed. Her vision, including visual acuity and field, were normal. Examinations of muscle tone, muscle power, deep tendon reflex and coordination were all unremarkable.

Additional file 1 Horizontal gaze palsy in this patient (video section). (AVI 2703 kb)


**Fig. 2:**
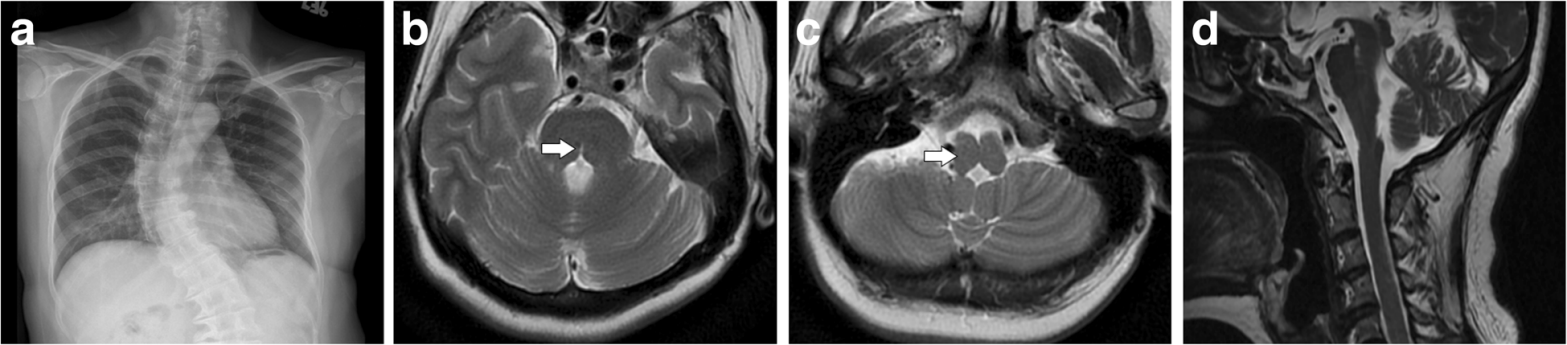
Main conventional imaging findings of this patient with horizontal gaze palsy with progressive scoliosis. **a** Chest X-ray showing S-shaped scoliosis of the thoracolumbar spine. **b** Axial T2-weighted brain magnetic resonance imaging showing a midline cleavage at the dorsal aspect of the pons (split pons sign) (arrow). **c** Butterfly configuration of the medulla (arrow). **d** Atrophic change of the pons in sagittal view

**Fig. 3:**
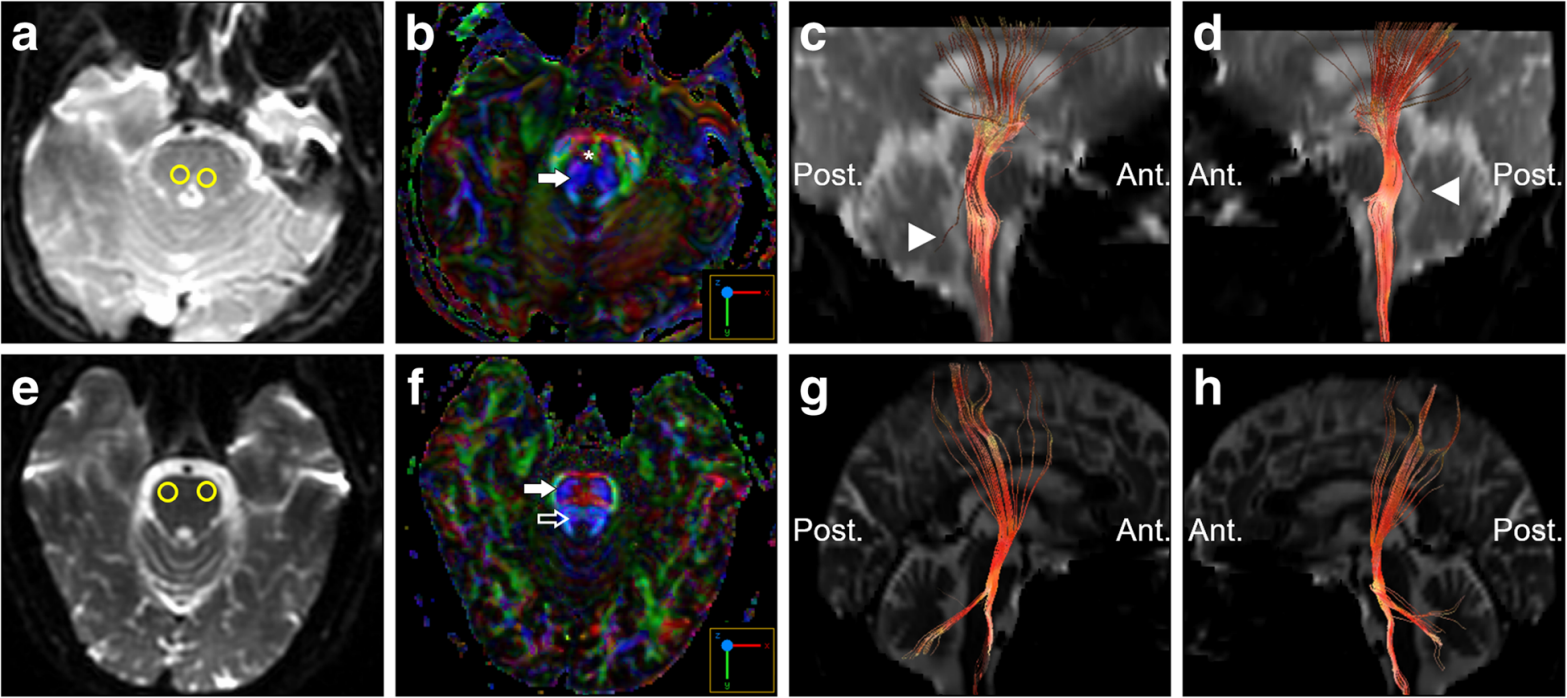
Diffusion tensor imaging showing an anatomical anomaly within the pons. **a, e** Selection of the seed regions of interest (35 mm^2^) in axial T2 weighted imaging. **b, f** Color-coded diffusion tensor imaging showing dorsal displacement of bilateral corticospinal tracts (solid arrows), malposition of the transverse pontine fibers (asterisk), and agenesis of the medial longitudinal fasciculus (hollow arrow in the healthy control). Tractography on the right (**c, g**) and left (**d, h**) side suggested agenesis of the pontocerebellar tracts (arrow heads) in the patients compared to the healthy control. Upper row: the patient; lower row: the age and sex-matched healthy control. Ant.: Anterior side; Post.: Posterior side

**Fig. 4:**
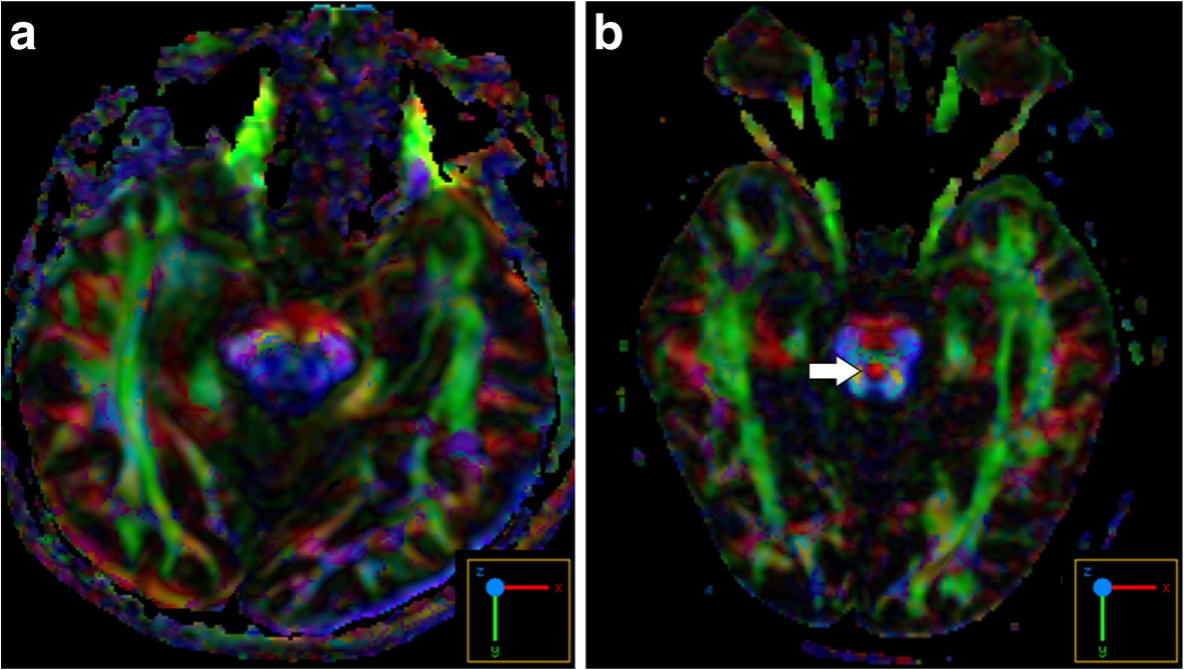
Color-coded diffusion tensor imaging showing absence of red dot in this patient (**a**) representing decussation of the superior cerebellar peduncle normally seen in the healthy control (arrow) (**b**)

**Fig. 5:**
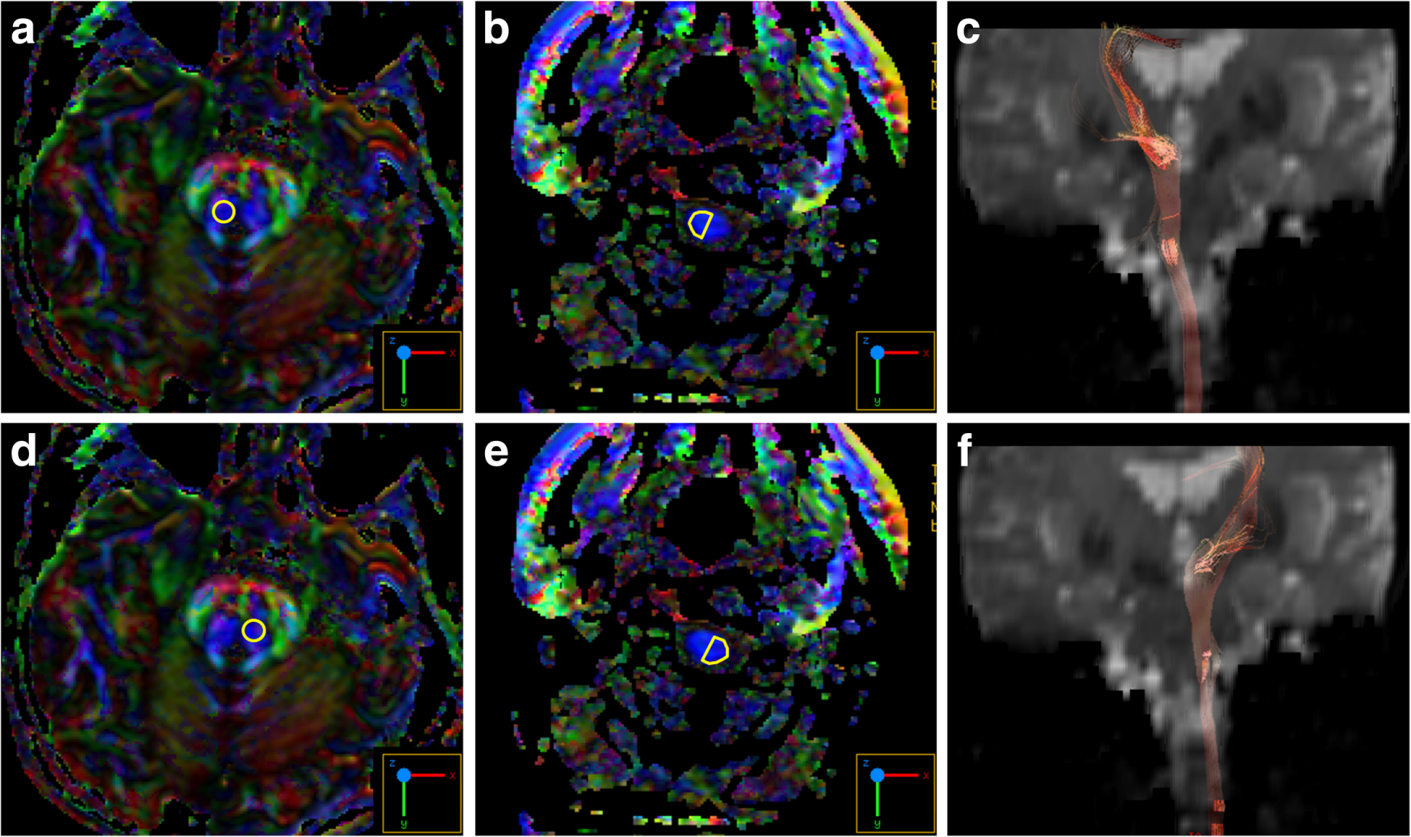
Tractography of the patient showing the uncrossed bilateral corticospinal tracts (**a**, **b**, **d**, **e** the seed regions selection; **c**, **f** the uncrossed bilateral corticospinal tracts). No crossed fibers were identified on placing the seed regions of the spinal cord contralateral to those within the pons

**Fig. 6:**
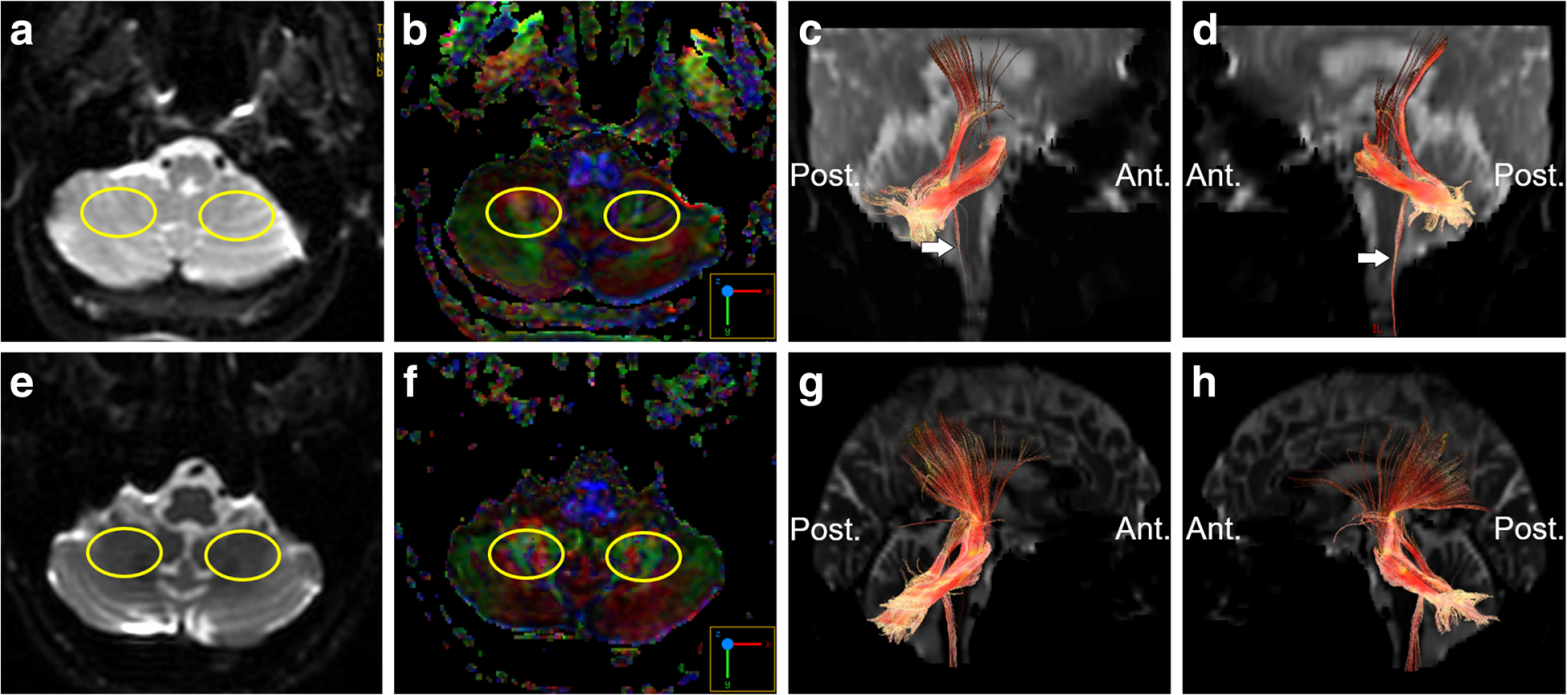
Tractography showing atrophic fibers within the inferior cerebellar peduncle (arrows). **a, e** Selection of the seed regions of interest (350 mm^2^) in axial T2 weighted imaging at approximately the same level. **b, f** Color-coded diffusion tensor imaging. **c, g** Tractography on the right-side. **d, h** Tractography on the left side. Upper row: the patient; lower row: the age and sex-matched healthy control. Ant.: Anterior side; Post.: Posterior side

**Fig. 7:**
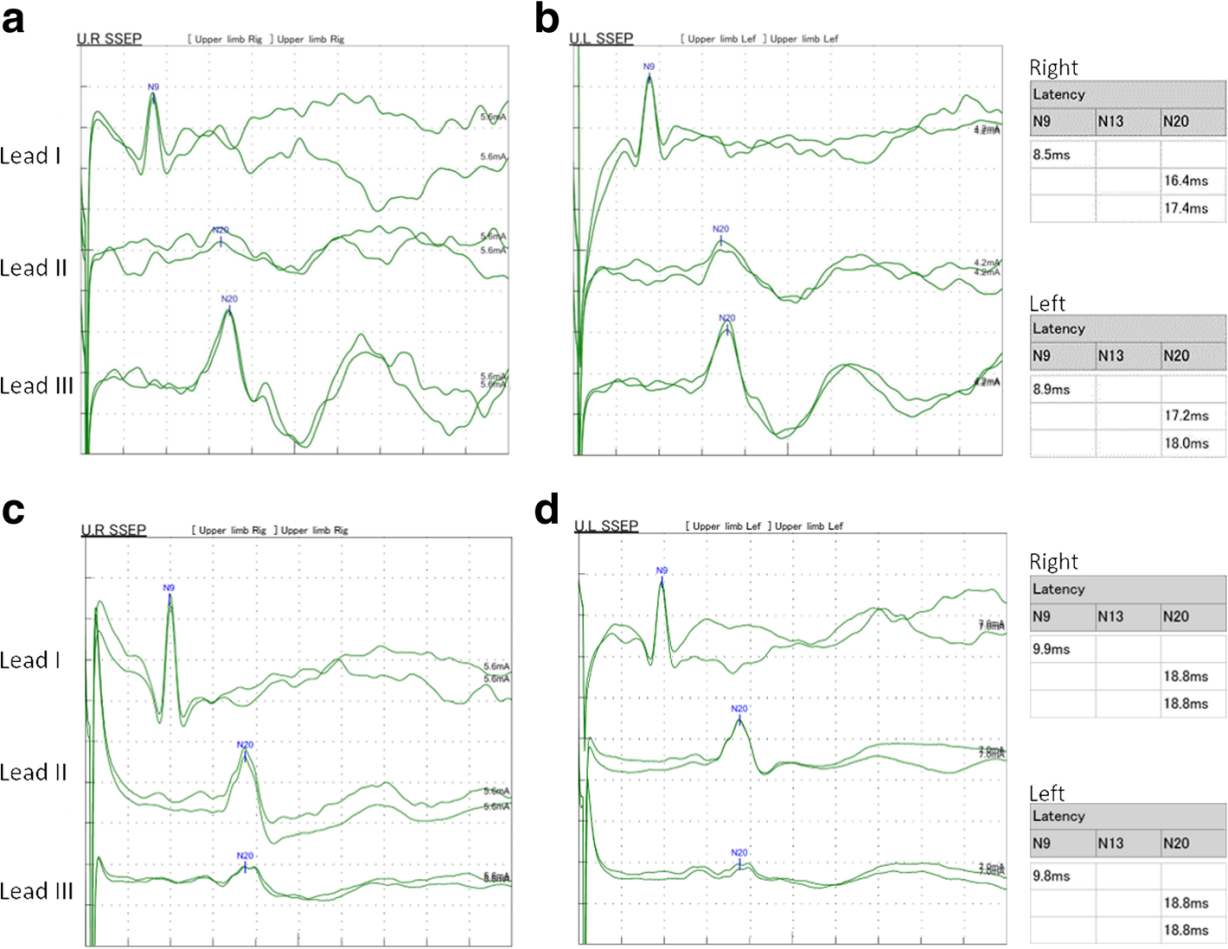
Somatosensory evoked potential studies comparing the patient and healthy control. Predominant signals of the cortex were detected ipsilateral to the stimulus in the patient (**a, b**), in contrast to predominant signals of the cortex contralateral to the stimulus in the healthy control (**c, d**). **a, c** the right-side stimulus. **b, d** the left-side stimulus. Lead I: ipsilateral Erb point, Lead II: contralateral cortex, Lead III: ipsilateral cortex

On reviewing her past medical history, she was born at term to healthy nonconsanguineous parents after a smooth pregnancy. No symptoms indicating central nervous system infection were noted during childhood. She had developed scoliosis since teenage, and X-rays revealed scoliosis of her thoracic and lumbar spine (Fig. 2a). She had received bracing treatment, but it had a limited benefit in halting progression of her thoracolumbar scoliosis. No diseases involving other systems were recorded, and there was no associated family history. Axial brain MRI showed a midline cleavage at the dorsal aspect of the pons (split pons sign) and butterfly configuration of the medulla (Fig. 2b, c), consistent with HGPPS. In sagittal view, the pons and medulla were dysmorphic with flattening of both anterior and posterior contours (Fig. 2d). DTI was performed using a 3.0-T MR unit (Discovery MR750, GE Medical Systems, Milwaukee, WI). The diffusion-sensitizing gradients were applied along 20 non-collinear directions with diffusion weighting factor b = 1000 s/mm^2^, plus one b = 0 image. The imaging parameters were: TR/TE = 8000/82 ms, matrix size = 128 × 128, field of view = 240 mm, slice thickness = 3 mm with no intersection gap, number of excitations = 2, number of slices = 67, scan time = 5 min and 58 s. The post-processing software Functool (GE Medical System, Milwaukee, WI) was used to generate fiber tracts. The seed points were placed in the brainstem based on T2-weighted images. The cutoff value for fractional anisotropy was 0.20 (default value). In color-coded DTI, the transverse pontine fibers (labeled in red by their latero-lateral connection) which separated the corticospinal tracts anteriorly from the medial lemniscus and medial longitudinal fasciculus posteriorly in the normal healthy control, were abnormally located anterior to the corticospinal tracts (Fig. 3b, f). The medial longitudinal fasciculus was compromised secondary to dorsal displacement of the bilateral corticospinal tracts (labeled in blue by their cranio-caudal orientation) (Fig. 3b, f). Interestingly, the pontocerebellar tracts were agenetic compared to the size in the normal healthy control (Fig. 3c, d, g, h). Another interesting color-coded DTI finding was absence of red dot at the level of the pontomesencephalic junction in our patient (Fig. 4). The red dot identified in the normal healthy control represents decussation of the superior cerebellar peduncle, presumably to be fibers normally oriented in a latero-lateral direction. Subsequent tractography revealed that the bilateral corticospinal tracts remained uncrossed at the level presumed to be the pyramidal decussation (Fig. 5). On tracing the fibers within the inferior cerebellar peduncle, the targeted fibers, deemed to be primarily dorsal spinocerebellar and vestibulocerebellar tracts, appeared to be smaller in size compared to the normal healthy control (Fig. 6). Interestingly, the cortical sensory responses showed abnormally reversed lateralization in the patient (Fig. 7). Specifically, although the latencies of all detectable responses were normal (Right N9 = 8.5 ms, N20 contralateral/ipsilateral to stimulus = 16.4 /17.4 ms; Left N9 = 8.9 ms, N20 contralateral/ipsilateral N20 = 17.2/18.0 ms; reference value N9 = 9 ms, N20 = 20 ms), a somatosensory evoked potential study revealed that the cortical sensory response was abnormally ipsilateral (Fig. 7).

## Discussion and conclusions

In this case report, we combined DTI/tractography and electrophysiological studies to reveal predominantly ipsilateral sensorimotor findings of HGPPS. The compromised tegmentum secondary to dorsal displacement of the corticospinal tracts and the poorly-developed pontocerebellar tracts and afferent fibers within the inferior cerebellar peduncle may be the main neuroanatomical anomalies responsible for the clinical presentations of HGPPS.

There are currently no autopsy reports of patients with HGPPS. However, incorporating advanced neuroimaging studies with clinical assessments may provide information to improve the understanding of its fundamental pathogenesis. The horizontal conjugate eye movement paralysis without esotropia or lateral rectus muscle atrophy in our case indicated that the lesion site was more likely to be at the medial longitudinal fasciculus rather than the abducens nuclei. Our color-coded DTI also corroborated this hypothesis, as the tegmentum containing the medial longitudinal fasciculus was expected to be compromised by dorsal displacement of bilateral corticospinal tracts. In the presence of profound reduction of midline crossing fibers within the brainstem, several DTI studies have further addressed the maldevelopment of fibers within the superior and middle cerebellar peduncles. The majority of DTI studies identified absent decussation of fibers from the superior cerebellar peduncles [5, 8]. Our color-coded DTI observation was also consistent with this anatomical feature. However, there is a considerable variation regarding the pontocerebellar tracts. Contrary to normal crossing of the fibers within the middle cerebellar peduncle identified by Avadhani et al. [8], the decussating pontine fibers were not visualized by Sicotte et al. [9]. On the other hand, Otaduy et al. described the findings indicative displacement of the pontocerebellar tracts [5]. Descriptions related to fibers passing through the inferior cerebellar peduncles are even more limited; isolated study has mentioned size reduction of the inferior cerebellar peduncles as an important component of butterfly configuration of the medulla [9]. Of note, the afferent fibers of both the pontocerebellar tracts and those within the inferior cerebellar peduncles were visualized to be smaller in size in our patient than in the normal control through our tractography. Such observation provided additional pathoanatomical information in HGPPS. Agenesis of the pontocerebellar tracts, as evidenced by both our color-coded DTI and tractography, suggests the possibility of impaired sensory input from the cortex. A recent DTI study suggested that the pontocerebellar tracts not only govern pure coordination of simple movement but also relay feed-forward and feed-backward connections due to its widespread cortical connectivity [10]. Therefore, it is possible that patients with HGPPS share a similar mechanism to patients with idiopathic scoliosis, whose abnormal sensorimotor network has been proposed to be part of the main pathogenesis [11]. Agenesis of fibers within the inferior cerebellar peduncles indicates a less competent state of the dorsal spinocerebellar and vestibulocerebellar tracts, thereby leading to spinal deformity by impairing proprioception signals from the spinal cord [12]. In other words, these fibers receive and convey proprioceptive information from the posterior column and vestibular nucleus to the cerebellum, and this process is thought to be critical as it mediates control signals back to the spinal cord to drive locomotion and regulate muscle tone. Several other hypothetical mechanisms have also been proposed to explain the progressive spinal deformity, including maldevelopment of the descending reticulospinal tracts [3], defects in other structures within the brainstem (e.g., the vestibular nucleus and medial longitudinal fasciculus) [1], and functional anomalies within the pontine reticular formation [7]. Although our DTI study could not exclude these possibilities, our electrophysiological studies support the primary mechanism of progressive scoliosis relying mainly on the brainstem rather than on the spinal cord itself. In addition, other studies have also mentioned heterogeneous ophthalmological findings with varying eyeball alignment and convergence performance [7]. This suggests that the corresponding brainstem lesions may involve more than the medial longitudinal fasciculus, and the possibility that a spectrum of abnormalities may be involved.

Various homozygous mutations throughout the human *ROBO3* gene located at chromosome 11q23–25 have been associated with HGPPS. The protein product of this novel gene is believed to specify the lateral position of longitudinal pathways [13] and direct cell migration [14]. Consistent with genetic studies, animal models using mice have demonstrated similar findings, suggesting the critical role of the *ROBO3* gene across species. Three cases with the clinical features of HGPPS have been reported to have hemiplegia ipsilateral to cerebral damage [15–17]. Our electrophysiological study and DTI findings also support that the uncrossed nature of both the pyramidal and proprioceptive sensory systems are due to *ROBO3* mutations in human neurodevelopment.

Patients with other conditions including Möbius syndrome and Duane retraction syndrome may also have similar clinical presentations of congenital horizontal gaze paresis. They frequently exhibit additional neurological signs related to agenesis of other cranial nuclei (e.g., facial nuclei) [18, 19], but not to have a spilt pons sign pathognomonic to HGPPS. Patients with congenital fibrosis of the extraocular muscles also commonly present with variable phenotypes (e.g., ptosis and infraducted globes) related to the maldevelopment of different subnuclei in isolation or combination [20]. Chronic progressive external ophthalmoplegia should also be considered in a differential diagnosis of ocular motility deficits. However, these patients frequently have variable clinical presentations combing ptosis, vision deficits, muscle weakness, and heart involvement due to its underlying mitochondrial dysfunction [21]. Congenital esotropia (or Ciancia syndrome) is less likely due to its presentation during early infancy [22]. We are aware that the interpretations of our current case would be limited by the lack of *ROBO3* genetics. However, we regard the diagnosis of HGPPS to be correct based onto classical presentations in this patient. Although our patients started to present her clinical symptoms since her childhood, the delayed age at diagnosis compared to the previous reports [5, 6, 8, 9] were likely due to rarity of HGPPS in clinical practice. A better understanding of the uncrossing nature of major pathway could facilitate clinicians’ awareness when dealing with HGPPS patients suffered from various brain insults [15–17].

This case report supports the hypothesis that maldevelopment of the tegmentum plays a crucial role in the pathogenesis of HGPPS. Horizontal gaze palsy could be explained by a compromised medial longitudinal fasciculus, whereas scoliosis could be due to agenesis of the pontocerebellar tracts and afferent fibers within the inferior cerebellar peduncles. In addition, the uncrossed nature of the majority of both the pyramidal and proprioceptive sensory systems was confirmed.

## Abbreviations

DTI: Diffusion tensor imaging
HGPPS: Horizontal gaze palsy with progressive scoliosis
MRI: Magnetic resonance imaging

## References

[1] Rossi A, Catala M, Biancheri R, Di Comite R, Tortori-Donati P (2004). MR imaging of brain-stem hypoplasia in horizontal gaze palsy with progressive scoliosis. AJNR Am J Neuroradiol.

[2] dos Santos AV, Matias S, Saraiva P, Goulao A (2006). MR imaging features of brain stem hypoplasia in familial horizontal gaze palsy and scoliosis. AJNR Am J Neuroradiol.

[3] Jen JC, Chan WM, Bosley TM, Wan J, Carr JR, Rub U (2004). Mutations in a human ROBO gene disrupt hindbrain axon pathway crossing and morphogenesis. Science.

[4] Pieh C, Lengyel D, Neff A, Fretz C, Gottlob I (2002). Brainstem hypoplasia in familial horizontal gaze palsy and scoliosis. Neurology.

[5] Otaduy MC, Leite Cda C, Nagae LM, Pinho Mda C, Bueno C, Reed UC (2009). Further diffusion tensor imaging contribution in horizontal gaze palsy and progressive scoliosis. Arq Neuropsiquiatr.

[6] Haller S, Wetzel SG, Lutschg J (2008). Functional MRI, DTI and neurophysiology in horizontal gaze palsy with progressive scoliosis. Neuroradiology.

[7] Bosley TM, Salih MA, Jen JC, Lin DD, Oystreck D, Abu-Amero KK (2005). Neurologic features of horizontal gaze palsy and progressive scoliosis with mutations in ROBO3. Neurology.

[8] Avadhani A, Ilayaraja V, Shetty AP, Rajasekaran S (2010). Diffusion tensor imaging in horizontal gaze palsy with progressive scoliosis. Magn Reson Imaging.

[9] Sicotte NL, Salamon G, Shattuck DW, Hageman N, Rub U, Salamon N (2006). Diffusion tensor MRI shows abnormal brainstem crossing fibers associated with ROBO3 mutations. Neurology.

[10] Keser Z, Hasan KM, Mwangi BI, Kamali A, Ucisik-Keser FE, Riascos RF (2015). Diffusion tensor imaging of the human cerebellar pathways and their interplay with cerebral macrostructure. Front Neuroanat.

[11] Domenech J, Garcia-Marti G, Marti-Bonmati L, Barrios C, Tormos JM, Pascual-Leone A (2011). Abnormal activation of the motor cortical network in idiopathic scoliosis demonstrated by functional MRI. Eur Spine J.

[12] Salamon N, Sicotte N, Drain A, Frew A, Alger JR, Jen J (2007). White matter fiber tractography and color mapping of the normal human cerebellum with diffusion tensor imaging. J Neuroradiol.

[13] Rajagopalan S, Vivancos V, Nicolas E, Dickson BJ (2000). Selecting a longitudinal pathway: Robo receptors specify the lateral position of axons in the Drosophila CNS. Cell.

[14] Wu W, Wong K, Chen J, Jiang Z, Dupuis S, Wu JY (1999). Directional guidance of neuronal migration in the olfactory system by the protein slit. Nature.

[15] Hosokawa S, Tsuji S, Uozumi T, Matsunaga K, Toda K, Ota S (1996). Ipsilateral hemiplegia caused by right internal capsule and thalamic hemorrhage: demonstration of predominant ipsilateral innervation of motor and sensory systems by MRI, MEP, and SEP. Neurology.

[16] Ng AS, Sitoh YY, Zhao Y, Teng EW, Tan EK, Tan LC (2011). Ipsilateral stroke in a patient with horizontal gaze palsy with progressive scoliosis and a subcortical infarct. Stroke.

[17] Yamada S, Okita Y, Shofuda T, Yoshioka E, Nonaka M, Mori K (2015). Ipsilateral hemiparesis caused by putaminal hemorrhage in a patient with horizontal gaze palsy with progressive scoliosis: a case report. BMC Neurol.

[18] Pedraza S, Gamez J, Rovira A, Zamora A, Grive E, Raguer N (2000). MRI findings in Mobius syndrome: correlation with clinical features. Neurology.

[19] Ozkurt H, Basak M, Oral Y, Ozkurt Y (2003). Magnetic resonance imaging in Duane's retraction syndrome. J Pediatr Ophthalmol Strabismus.

[20] Engle EC, Leigh RJ (2002). Genes, brainstem development, and eye movements. Neurology.

[21] Zeviani M, Di Donato S (2004). Mitochondrial disorders. Brain.

[22] Helveston EM (1993). 19th annual frank Costenbader lecture--the origins of congenital esotropia. J Pediatr Ophthalmol Strabismus.

